# Chalcone Derivatives 4′-Amino-1-Naphthyl-Chalcone (D14) and 4′-Amino-4-Methyl-1-Naphthyl-Chalcone (D15) Suppress Migration and Invasion of Osteosarcoma Cells Mediated by p53 Regulating EMT-Related Genes

**DOI:** 10.3390/ijms19092838

**Published:** 2018-09-19

**Authors:** Viviane Seba, Gabriel Silva, Mariana Bastos dos Santos, Seung Joon Baek, Suzelei de Castro França, Ana Lúcia Fachin, Luis Octavio Regasini, Mozart Marins

**Affiliations:** 1 Biotechnology Unit, University of Ribeirão Preto, Ribeirão Preto, SP CEP 14096-900, Brazil; vivianeseba@gmail.com (V.S.); biel-189@hotmail.com (G.S.); sfranca@unaerp.br (S.d.C.F.); afachin@unaerp.br (A.L.F.); 2 Laboratory of Green and Medicinal Chemistry, Department of Chemistry and Environmental Sciences, Institute of Biosciences, Humanities and Exact Sciences, São Paulo State University (UNESP), São José do Rio Preto, SP CEP 15054-000, Brazil; mariana19bsantos@gmail.com; 3 Laboratory of Signal Transduction, College of Veterinary Medicine and Research Institute for Veterinary Science, Seoul National University, Seoul 08826, Korea; baeksj@snu.ac.kr; 4 Medicine School, University of Ribeirão Preto, Ribeirão Preto, SP CEP 14096-900, Brazil

**Keywords:** osteosarcoma, chalcones, p53, migration, invasion, epithelial-mesenchymal transition

## Abstract

Osteosarcoma (OS) is a primary malignant bone tumor that mainly affects children, adolescents, and young adults. The inhibition of metastasis is a main strategy of OS therapy since the development of metastatic disease due to drug resistance remains the most important cause of death from this cancer. Considering the severe side effects of current OS chemotherapy, the identification of anti-metastatic drugs with reduced toxicity is of great interest. Chalcones are polyphenols with a basic structure consisting of an α-, β-unsaturated carbonyl system linking two aryl rings. These compounds exhibit anticancer activity against a variety of tumor cell lines through multiple mechanisms, including the regulation of the tumor-suppressor protein p53 and its target genes. An important process regulated by p53 is epithelial-mesenchymal transition (EMT), which facilitates tumor metastasis by conferring migratory and invasive properties to cancer cells. The activation of p53 can revert EMT and reduce migration and invasion. This study aimed to examine the inhibitory effects of two 4′-aminochalcones on the migration/invasion of the U2OS (p53+/+) and SAOS-2 (p53−/−) OS cell lines as well as the underlying molecular mechanisms. Cell viability was examined by MTT assay. Transwell assays were used to evaluate the migratory and invasive ability of the cells. The two 4′-aminochalcones showed low capacity to inhibit the viability of OS cells independent of p53 status, but preferentially suppressed the migration of U2OS cells and of a SAOS-2 cell line expressing p53. Invasion was strongly inhibited by both chalcones independent of p53 status. RT-PCR, zymography, and Western blot were used to study the expression of matrix metalloproteinases and EMT markers after treatment with the chalcones. The results indicated that the 4′-aminochalcone-induced antimigratory and anti-invasive effects are potentially associated with the inhibition of extracellular matrix (ECM) enzymatic degradation in OS cells and with the modulation of EMT genes. These effects probably result from the induced increase of p53 protein expression by the two chalcones. In conclusion, chalcones D14 and D15 have potential anti-metastatic activity mediated by p53 that can be exploited for OS treatment.

## 1. Introduction

Osteosarcoma is an extremely malignant bone tumor variety whose peak incidence occurs between 10 and 20 years of age [1]. Despite advances in osteosarcoma treatment which include local surgical resection and multidrug chemotherapy, this type of tumor shows a high tendency for local invasion and distant metastasis, a fact that decreases the long-term survival rate of metastatic patients to 20–30% [2]. The lung is the most common site of metastasis of osteosarcoma and lung metastases usually lead to death within a year [3].

Cell migration and invasion are two critical cellular processes for the metastasis of cancer cells from the primary tumor to distant sites. These processes involve the degradation of the extracellular matrix (ECM) and epithelial-mesenchymal transition (EMT). The breakdown of the ECM by proteolytic enzymes, such as matrix metalloproteinases (MMPs), can facilitate the migration and invasion of tumor cells toward the vascular/lymphatic system, ultimately triggering metastasis [4,5]. More than 20 MMPs have been described and MMP-2 and MMP-9 are strongly associated with tumor spread and invasiveness [6]. A high production of MMP-2 and MMP-9 increases the migration and invasion of tumor cells and is associated with a poor prognosis in cancer patients [7].

Tumor cells undergo changes in their transcriptome during EMT that control morphological and biochemical modifications, including a decrease in epithelial proteins (e.g., E-cadherin) and an increase in mesenchymal proteins such as vimentin and N-cadherin. These alterations enhance the ability of tumor cells to migrate, invade, and cause metastasis [8]. An important regulator of EMT is the p53 tumor suppressor protein. During metastasis, the interaction of p53 with transcription factors such as *Snail* and *Slug* regulates the expression of genes vital to the onset and progression of EMT [9,10]. Studies have shown that the activation of p53 can promote the reversal of mesenchymal cells to the epithelial cell phenotype and the subsequent reduction of migration and invasion [11].

Our research group has been working to identify small molecules that can be used as metastasis inhibitors through the induction of p53 [12,13]. Prominent among these are chalcones, a group of polyphenolic compounds with an alpha-beta unsaturated ketone core moiety that exerts cytotoxic activity against different tumor cell lines through mechanisms such as cell cycle disruption, the inhibition of angiogenesis, and the induction of apoptosis [14,15,16,17]. In previous works using U2OS osteosarcoma cells and other tumor cell lines, we found that trans-chalcone has apoptotic activity mediated by p53 activation, in addition to regulating genes involved in cell migration and invasion [18]. These data prompted us to screen a library of 68 chalcones to identify new molecules with potential to inhibit proliferation and migration in osteosarcoma cells. We found two 4′-aminochalcones that exhibited low cytotoxicity, but a great capacity to inhibit migration in osteosarcoma cells expressing p53 [13].

In the present study, we further investigated the inhibitory effects of these 4′-aminochalcones on osteosarcoma migration/invasion. The results revealed that the underlying molecular mechanisms of these effects involve the upregulation of p53 and the downregulation of MMPs and EMT.

## 2. Results

### 2.1. 4′-Aminochalcones Poorly Inhibit Viability of Osteosarcoma and Normal Cells

**Figure 1:**
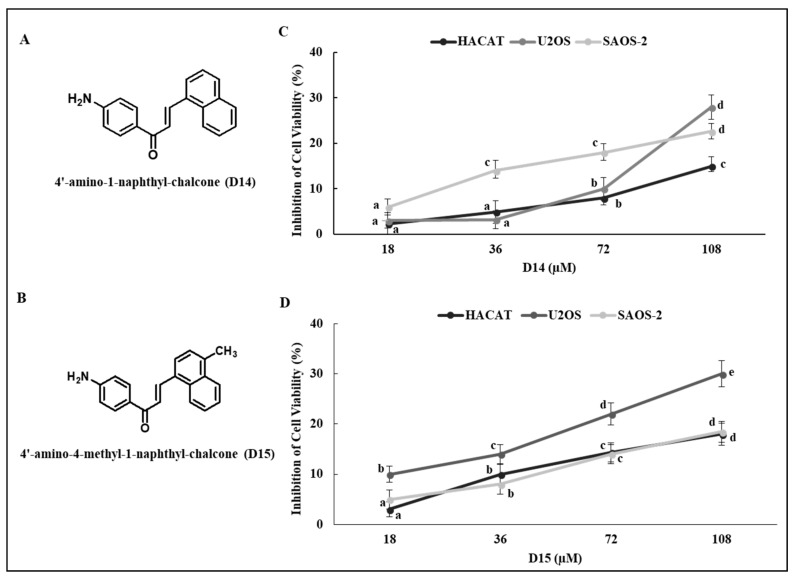
Inhibition of cell viability by D14 and D15 evaluated by MTT assay. (**A**,**B**) Chemical structure of D14 and D15. (**C**,**D**) Osteosarcoma cells (U2OS and SAOS-2) and human keratinocytes (HaCaT) were treated with the indicated concentrations of D14 and D15 for 24 h (control: cells treated with 0.1% DMSO). The values are expressed as means ± standard deviation of three individual experiments. Means followed by the same letter are not significantly different (*p* < 0.05) according to Tukey’s HSD test.

To investigate the effects of 4′-amino-1-naphthyl-chalcone (D14) and 4′-amino-4-methyl-1-naphthyl-chalcone (D15) (Figure 1A,B) on osteosarcoma (U2OS and SAOS-2) and normal (HaCaT) cell viability, we treated cells with the chalcones at concentrations of 18, 36, 72, and 108 µM for 24 h. The two 4′-aminochalcones showed a low capacity to inhibit the viability of osteosarcoma (<30% inhibition) and normal cells (<20% inhibition) even at 108 µM (Figure 1C,D). We used 54 µM as the highest concentration in all subsequent experiments to examine the anti-metastatic properties of the 4′-aminochalcones D14 and D15.

### 2.2. 4′-Aminochalcones Decrease Osteosarcoma Cell Migration and Invasiveness

**Figure 2:**
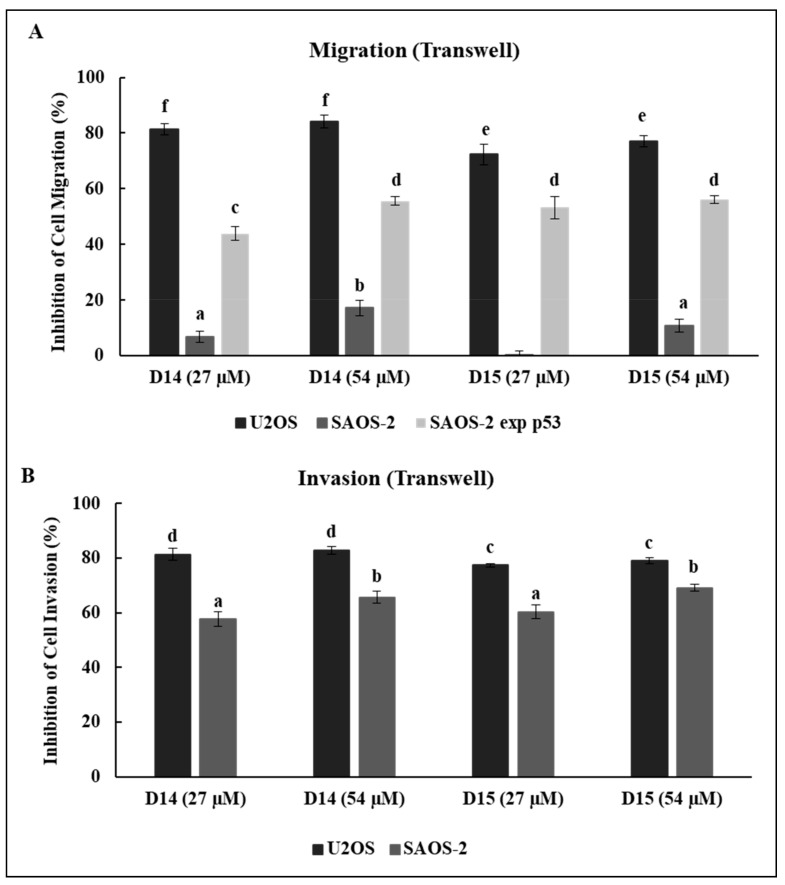
Effects of D14 and D15 on the migratory and invasive abilities of osteosarcoma cells. (**A**) Migration of osteosarcoma cells in Transwells. (**B**) Invasion of osteosarcoma cells in Transwells pre-coated with Matrigel. For migration and invasion, cells in serum-free medium were placed in the top chamber of the Transwell. Complete medium (10% serum) containing D14 and D15 at the indicated doses was added to the lower chamber. After 24  h, the cells that had migrated or invaded through the membrane were stained and quantified. The results are expressed as means ± standard deviation of three individual experiments. Means followed by the same letter are not significantly different (*p* < 0.05) according to Tukey’s HSD test.

To examine the effects of 4′-aminochalcones on osteosarcoma cell migration and invasion, we used uncoated and Matrigel-coated Transwells, respectively. As shown in Figure 2A, chalcone D14 markedly decreased the migration of U2OS cells (>80%) but had a poor effect on SAOS-2 cells (<18%). D15 presented similar effects, reducing the migration of U2OS and SAOS-2 by more than 70% and by less than 11%, respectively. However, in SAOS-2 expressing p53 stable clone (SAOS-2 exp p53), chalcones D14 and D15 reduced cell migration by more than 44%. As can be seen in Figure 2B, both 4′-aminochalcones strongly reduced the invasion (>75%) of U2OS and SAOS-2 cells, but this inhibition was slightly higher for the U2OS cell line. Taken together, the 4′-aminochalcones appeared to be most effective in p53-expressing osteosarcoma cells.

### 2.3. 4′-Aminochalcones Attenuate the Expression and Proteolytic Activity of MMP-2 and MMP-9 in Osteosarcoma Cells

**Figure 3:**
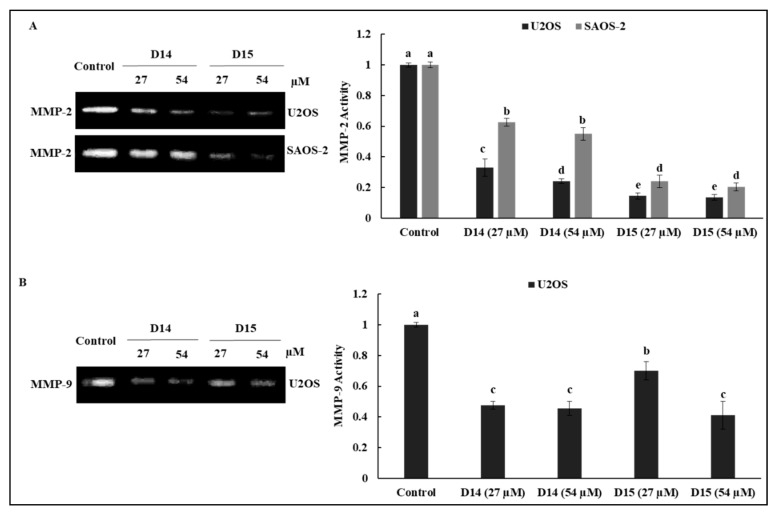
Effects of D14 and D15 on the proteolytic activity of matrix metalloproteinases (MMP)-2 and MMP-9 in osteosarcoma cells. U2OS and SAOS-2 cells were treated with D14 and D15 in serum-free medium at the indicated concentrations for 24 h. Next, the supernatant (conditioned medium) was collected and subjected to gelatin zymography to analyze the activity of secreted MMP-2 (**A**) and MMP-9 (**B**). Quantitative results of three independent experiments are expressed as means ± standard deviation. Means followed by the same letter are not significantly different (*p* < 0.05) according to Tukey’s HSD test.

Gelatin zymography was performed to evaluate whether the 4′-aminochalcone-induced inhibition of migration and invasion was related to the downregulation of MMP-2 and MMP-9 proteolytic activities. As shown in Figure 3, chalcones D14 and D15 decreased MMP-2 activity in osteosarcoma cells, but this inhibition was more pronounced in p53-expressing cells (U2OS), especially after treatment with chalcone D14. The chalcones also repressed MMP-9 activity in U2OS cells, but we did not detect MMP-9 activity in SAOS-2 cells.

**Figure 4:**
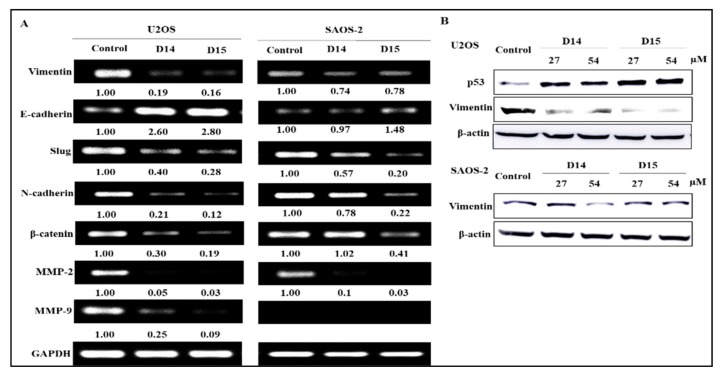
Effects of D14 and D15 on the expression of MMPs and epithelial-mesenchymal transition (EMT)-related genes. (**A**) Osteosarcoma cells were treated with 54 µM of D14 and D15 in serum-free medium for 24 h (control: cells treated with 0.1% DMSO). Next, total RNA was isolated and subjected to conventional RT-PCR. Representative gel and densitometry analyses are shown. The image intensities of the bands were normalized against the intensity of the GAPDH band. (**B**) Analysis of p53 and vimentin protein expression in osteosarcoma cells treated with D14 and D15 at the indicated concentrations for 24 h. After treatment, cell lysates (30 µg of total protein) were obtained and subjected to Western blot analysis using anti-p53, anti-vimentin, and anti-β-actin antibody.

RT-PCR was performed to further elucidate the inhibitory effects of the 4′-aminochalcones on MMPs. Chalcones D14 and D15 significantly decreased mRNA expression of *MMP-2* in U2OS and SAOS-2 cells, and once more chalcone D14 showed a stronger effect on the U2OS cell line (Figure 4A). Chalcones D14 and D15 also downregulated MMP-9 mRNA levels in U2OS cells. However, MMP-9 mRNA was not detected in the SAOS-2 cell line. These results indicate that the 4′-aminochalcone-induced antimigratory and anti-invasive effects are potentially associated with the inhibition of ECM enzymatic degradation in osteosarcoma cells.

### 2.4. 4′-Aminochalcones Regulate EMT Gene Expression in Osteosarcoma Cells

We examined the expression of EMT-related genes by RT-PCR to support the antimigratory and anti-invasive effects of the 4′-aminochalcones on osteosarcoma cells. As shown in Figure 4A, chalcones D14 and D15 strongly upregulated E-cadherin and downregulated vimentin mRNA levels in U2OS cells and showed no effect or only a slight effect in SAOS-2 cells. The two 4′-aminochalcones also decreased *Slug* gene expression in both osteosarcoma cell lines. Chalcone D14 significantly decreased N-cadherin and β-catenin mRNA levels only in U2OS cells. On the other hand, D15 markedly inhibited these genes in U2OS and SAOS-2 cells. The effect of the 4′-aminochalcones on vimentin expression was confirmed by Western blotting (Figure 4B). We found that D14 and D15 significantly suppressed vimentin protein levels only in U2OS cells, consistent with the effect observed in the RT-PCR assay. As shown in Figure 4B, chalcones D14 and D15 also strongly increased p53 protein expression in U2OS cells.

## 3. Discussion

Osteosarcoma is the most recurrent malignant bone tumor, which is characterized by a highly metastatic potential. The molecular mechanisms underlying the metastasis of osteosarcoma are multifaceted. Studies have shown that ECM degradation mediated by MMP-2 and MMP-9 plays a critical role in the motility and invasiveness of osteosarcoma cells [19,20,21]. The expression of MMP-2 and MMP-9 is upregulated in osteosarcoma tissue and is associated with pulmonary metastasis and lower overall survival in patients with osteosarcoma [22,23]. The progression and metastasis of osteosarcoma have also been linked to the EMT process [24]. The upregulation of vimentin and N-cadherin (mesenchymal markers) expression and the downregulation of E-cadherin (epithelial marker) enhance the ability of osteosarcoma cells to migrate and invade [25,26]. Furthermore, the establishment of osteosarcoma lymph node and lung metastases is related to the inactivation of the wild-type function of the p53 tumor-suppressor protein [27,28,29]. The delivery of wild-type p53 to osteosarcoma cells inhibits migration and invasion in vitro and suppresses osteosarcoma tumor growth and lung metastases in vivo [30,31,32]. Wild-type p53 is also able to block the EMT process and to reduce the metastatic potential of tumor cells [33,34]. These data suggest that the restoration of wild-type p53 function could be a therapeutic approach for osteosarcoma.

Many investigators, including us, have shown that chalcones exert anticancer activity against different cancer cell lines through mechanisms such as cell cycle disruption, the inhibition of angiogenesis, tubulin polymerization, the induction of apoptosis, the blockade of the NF-κB signaling pathway, and the induction of p53 protein [17,18,35]. We recently screened a small semi-synthetic chalcone library and found chalcones with potential to inhibit the migration of osteosarcoma cells [13]. In this study, we investigated in detail the antimigratory and anti-invasive effects of 4′-amino-1-naphthyl-chalcone (D14) and 4′-amino-4-methyl-1-naphthyl-chalcone (D15) on osteosarcoma cells and elucidated the molecular mechanism underlying these effects.

**Figure 5:**
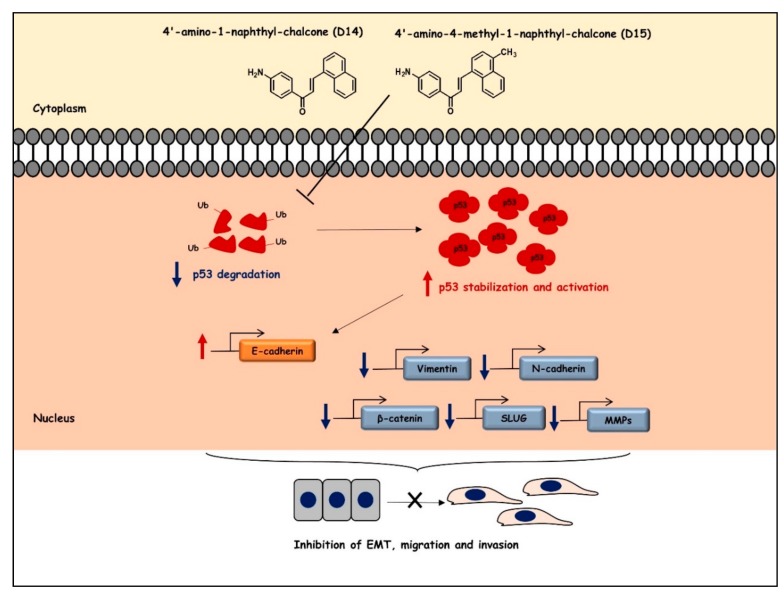
A proposed model for the action of chalcones D14 and D15 in osteosarcoma cells. D14 and D15 upregulated p53 protein levels, thus regulating metalloproteinases (MMPs) and EMT-related genes and promoting the inhibition of cell migration and invasion.

We observed that these 4′-aminochalcones have a low capacity to suppress the cell viability of the osteosarcoma cell lines U2OS and SAOS and of the non-tumor cell line HaCaT (Figure 1). Moreover, they do not induce apoptosis in osteosarcoma cells (data not shown). On the other hand, these 4′-aminochalcones demonstrated great potential as inhibitors of migration and invasion of osteosarcoma cells (Figure 2), especially in p53-expressing cells (U2OS). These effects seem to be related, at least in part, to the inhibition of the expression and proteolytic activity of MMP-2 and MMP-9 mediated by these 4′-aminochalcones (Figure 3). MMPs can facilitate the detachment of cells from the primary tumor, thus allowing these tumor cells to spread to distant sites, forming metastases. In osteosarcoma, studies indicated that the repression of MMP-2 and MMP-9 decreases the ability of osteosarcoma cells to migrate and invade [36,37]. Thus, the repression of MMPs might be an early target for preventing osteosarcoma metastasis and strengthening the anti-metastatic potential of the 4′-aminochalcones D14 and D15. Furthermore, we performed RT-PCR analysis to evaluate the regulation of EMT-related genes mediated by the 4′-aminochalcones D14 and D15. We found that these chalcones upregulate *E-cadherin* gene expression in osteosarcoma cells carrying the *TP53* gene (Figure 4A). A low expression of E-cadherin is associated with cancer metastasis; therefore, the recovery of E-cadherin expression can inhibit the EMT process and reduce the metastatic potential of cancer cells [38,39]. We also observed that D14 and D15 downregulate *vimentin*, *N-cadherin*, *Slug*, and *β-catenin* gene expression, especially in p53-expressing osteosarcoma cells. A high expression of these genes is associated with cancer metastasis and poor prognosis [40,41,42,43]. On the other hand, the inhibition of *vimentin*, *N-cadherin*, *Slug*, and *β-catenin* can reduce invasion, migration, and metastases development [44,45,46,47]. SLUG is a repressor of *E-cadherin* and studies have shown that SLUG overexpression in osteosarcoma downregulates the expression of *E-cadherin* [48]. Thus, it is likely that the induction of *E-cadherin* caused by the 4′-aminochalcones is mediated by *Slug* repression. A high expression of N-cadherin strongly increases the transcriptional activity of β-catenin and upregulates *MMP-9* expression in oral squamous cell carcinoma cells [49]. The knockdown of *β-catenin* dramatically inhibits invasion by downregulating MMP-2 and MMP-9 activity [50]. These data suggest that the inhibition of MMPs caused by D14 and D15 may be related to the repression of β-catenin, while the inhibitory effect on β-catenin is mediated by the inhibition of N-cadherin induced by these chalcones. Western blot analysis was performed to further confirm the downregulation of vimentin mediated by the 4′-aminochalcones D14 and D15. We found that these chalcones reduce vimentin protein levels (Figure 4B), indicating that they inhibit vimentin at the transcriptional and post-transcriptional levels. In general, D14 and D15 showed greater effects of inhibiting migration and regulating MMPs and EMT in p53-expressing osteosarcoma cells (U2OS), indicating the participation of p53 in these effects. To support these findings, we analyzed by Western blot the potential of D14 and D15 to increase p53 protein expression. D14 and D15 strongly increased p53 protein levels in U2OS cells (Figure 4B), reinforcing the idea that p53 may play a role in the action of these chalcones. Studies showed that p53 activation can promote the upregulation of E-cadherin and downregulation of β-catenin, SLUG, vimentin, and N-cadherin, consequently suppressing cancer cell growth and metastasis [51,52,53,54,55,56]. These data further support that these 4′-aminochalcones can inhibit EMT, migration, and invasion, which is mediated at least in part by the induction of p53 (Figure 5).

In this study, we found that the 4′-aminochalcones D14 and D15 reduce cell migration and invasion in osteosarcoma cells. This effect is more potent in p53-expressing cells. Furthermore, D14 and D15 increase p53 protein expression, decrease the expression and activity of MMP-2 and MMP-9, and downregulate EMT. All of these targets play an important role in cancer cell migration and invasion. Taken together, our data point to a potential anti-metastatic activity of D14 and D15 in osteosarcoma and provide the rationale for further in vivo studies in animal models to confirm these findings.

## 4. Materials and Methods

### 4.1. Cell Culture and Chemicals

The human osteosarcoma cell lines U2OS (p53 wt) and SAOS-2 (p53 null) and a human keratinocyte cell line (HaCaT) were purchased from the Rio de Janeiro Cell Bank (BCRJ, Federal University of Rio de Janeiro, Rio de Janeiro, Brazil). U2OS and SAOS-2 cells were grown in McCoy’s 5A medium (Sigma-Aldrich^®^, St. Louis, MO, USA), while HaCaT cells were grown in RPMI medium (Sigma-Aldrich^®^), supplemented with 10% fetal bovine serum (FBS), 100 U/mL penicillin, and 100 µg/mL streptomycin (Sigma-Aldrich^®^). The cells were cultured at 37 °C in a humidified atmosphere of 5% CO_2_ for all experiments. Chalcones D14 and D15 (Figure 1A) were provided by Dr. Luis Octávio Regasini (Department of Chemistry and Environmental Chemistry, São Paulo State University, São Paulo, Brazil).

### 4.2. Cell Viability Assay

The viability of the osteosarcoma cell lines was analyzed by MTT assay. In short, osteosarcoma cells were seeded at a concentration of 10,000 cells/well in 96-well culture plates in four replicates and incubated overnight. Next, the cells were treated with 0, 5, 10, 20, and 30 of D14 and D15 for 24 h. DMSO was used as a solvent at a final concentration of 0.1%, which is considered non-toxic for the cells. After treatment, the medium was replaced with fresh medium, 20 µL of a solution of MTT (3-(4,5-dimethylthiazol-2-yl)-2,5-diphenyltetrazolium bromide) (Sigma-Aldrich^®^) (5 mg/mL) was added to each well, and the plates were incubated for 3 h at 37 °C. Finally, cell viability was quantified by the detection of absorbance (550 nm) in a microplate reader (MultiSkan FC, Thermo Scientific, Waltham, MA, USA).

### 4.3. In Vitro Cell Migration and Invasion Assay

Transwell assays were performed to investigate the effect of D14 and D15 on the migration and invasion abilities of osteosarcoma cells. For the migration assay, 2 × 10^5^ cells resuspended in 200 µL serum-free medium were placed into the upper chamber of 24-well Transwell plates with a pore size of 8 µm (Corning^®^, Kennebunk, ME, USA). Next, 750 µL of culture medium containing different concentrations of D14 and D15 was added to the lower chamber together with a chemoattractant (10% FBS) and the plates were incubated for 24 h at 37 °C. Non-migrated cells on the upper side of the inserts were completely removed by swabbing, while the migrated cells attached to the bottom side of the Transwell membrane were fixed with paraformaldehyde and stained with Wright’s Giemsa solution [12]. For the invasion assay, the Transwell membranes were pre-coated with 0.75 mm of Matrigel (Corning^®^) according to the manufacturer’s instructions, and the same steps as in the migration assay were performed. Finally, cells that migrated or invaded were destained with 100 µL of 33% acetic acid. The destaining solution was collected and absorbance was measured at 490 nm in a microplate reader (MultiSkan FC, Thermo Scientific, Waltham, MA, USA). The inhibition of migration or invasion was calculated as follows: inhibition (%) = [1 − (absorbance of treated group/absorbance of control group)] × 100. The data represent the average ± SD of three independent experiments.

### 4.4. Gelatin Zymography

The activity of MMP-2 and MMP-9 was measured by gelatin zymography. Briefly, osteosarcoma cells were cultured in 6-well plates (5 × 10^5^ cells/well), followed by treatment with different concentrations of D14 and D15 in serum-free medium for 24 h. Appropriate volumes of the supernatant (conditioned medium) for each sample were collected and separated by 0.1% gelatin–7% SDS-PAGE electrophoresis. Next, the gels were soaked three times (30 min each) in 2.5% Triton X-100 at room temperature and incubated in reaction buffer (10 mM CaCl_2_, 40 mM Tris-HCl, and 0.01% NaN_3_, pH 8.0) for 18 h at 37 °C. The gels were rinsed with distilled water, stained with Coomassie brilliant blue R-250, and destained with methanol/acetic acid solution for 1 h at room temperature. Gelatinolytic activity appearing as clear bands was quantified by densitometry using ImageJ software (ImageJ2, National Institutes of Health, Bethesda, MD, USA).

### 4.5. Reverse Transcription-Polymerase Chain Reaction (RT-PCR)

Conventional RT-PCR was used to analyze MMPs and EMT-related gene expression. Briefly, osteosarcoma cells were cultured in 6-well plates (6.5 × 10^5^ cells/well), followed by treatment with different concentrations of D14 and D15 in serum-free medium for 24 h. Next, the cells were treated with 0, 10, 20, and 40 µL of CH-5 in serum-free medium for 24 h. Following treatment, total RNA was isolated using the ReliaPrep™ RNA Miniprep Systems (Promega, Madison, WI, USA). Next, 1 µg of RNA was used for the synthesis of cDNA using the GoScript Reverse Transcriptase kit (Promega). PCR was carried out using GoTaq^®^ Green Master Mix (Promega) with human primers as follows: MMP-2 Fwd 5′-ttccccttcttgttcaatgg-3′ and Rev 5′-atttgttgcccaggaaagtg-3′; MMP-9 Fwd 5′-ttgacagcgacaagaagtgg-3′ and Rev 5′-gccattcacgtcgtccttat-3′; Vimentin Fwd 5′-tgtccaaatcgatgtggatgtttc-3′ and Rev 5′-ttgtaccattcttctgcctcctg-3′; E-cadherin Fwd 5′-tgcccagaaaatgaaaaagg-3′ and Rev 5′-gtgtatgtggcaatgcgttc-3′; N-cadherin Fwd 5′-gacaatgcccctcaagtgtt-3′ and Rev 5′-ccattaagccgagtgatggt-3′; SLUG Fwd 5′-gagcatacagccccatcact-3′ and Rev 5′-gggtctgaaagcttggactg-3′; β-catenin Fwd 5′-gaaacggctttcagttgagc-3′ and Rev 5′-ctggccatatccaccagagt-3′; and GAPDH Fwd 5′-gaccacagtccatgccatcact-3′ and Rev 5′-tccaccaccctgttgctgtag-3′. The thermal cycling conditions were as follows: initial denaturation at 94 °C for 2 min, followed by 25–30 cycles at 94 °C for 30 s, 60 °C for 30 s, and 72 °C for 30 s, and a final extension at 72 °C for 5 min. The PCR products were electrophoresed on 1.5% agarose gel and photographed under UV light. The intensity of bands was analyzed by densitometry using the GAPDH band (constitutively expressed gene) as a reference.

### 4.6. Western Blot

Osteosarcoma cells were cultured in 6-well plates (6.5 × 10^5^ cells/well), followed by treatment with different concentrations of D14 and D15 in serum-free medium for 24 h. Next, cells were harvested in RIPA buffer supplemented with proteinase inhibitors and subjected to sonication (three times for 5 s each) and the cell lysate was centrifuged at 13,000× *g* for 15 min at 4 °C. The supernatant was collected, and the protein concentration was determined using the Pierce BCA Protein Assay Reagent (Thermo Scientific, Rockford, IL, USA). Amounts of total proteins (30 µg) were separated on 10% SDS-PAGE gel and transferred onto Amersham^TM^ Protran^TM^ 0.45-µm NC nitrocellulose membranes (GE Healthcare, Little Chalfont, UK). The membranes were blocked in Tris-buffered saline with Tween 20 (TBST) (25 mM Tris, 3 mM KCI, 0.14 M NaCl, and 0.05% Tween 20) containing 5% nonfat milk for 1 h at room temperature. Subsequently, the membranes were incubated in TBST–5% nonfat milk containing the primary antibodies (1:1000) overnight at 4 °C. After washing with TBST, the membranes were incubated with horseradish peroxidase-conjugated secondary antibodies diluted in TBST–5% nonfat milk (1:10,000) for 1 h and washed several times. The proteins were detected by chemiluminescence using the ECL Prime WB Detection Reagent (GE Healthcare) in an Image Quant LAS 500 luminescence analyzer (GE Healthcare).

### 4.7. Expression Vector and Transfection

The full-length p53 expression vector was generously provided by Dr. Seung Baek (Seoul National University, Seoul, Korea). The transfection was carried out using Lipofectamine 3000 Reagent (Thermo Scientific, Rockford, IL, USA) according to the manufacturer’s protocol. SAOS-2 cells (p53 null) grown in 6-well plates were transfected with 5 µg of p53 expression vector for 48 h. After transfection, SAOS-2 cells expressing p53 stable clones were selected in culture medium supplemented with 500 μg/mL G418 for 2 weeks.

### 4.8. Statistical Analysis

Statistical analysis was carried out using one-way ANOVA followed by Tukey’s HSD test. The results were considered statistically significant if * *p* < 0.05, ** *p* < 0.01, and *** *p* < 0.001.
